# Mantle cell lymphoma presenting as a pelvi-ureteric junction obstruction: a case report

**DOI:** 10.1186/1752-1947-7-105

**Published:** 2013-04-16

**Authors:** Ishvar Naranji, Rhana H Zakri, Thomas Liston

**Affiliations:** 1 Department of Urology, Worthing Hospital, West Sussex BN11 2DH, UK

## Abstract

**Introduction:**

Mantle cell lymphoma is one of the several subtypes of non-Hodgkin’s lymphoma. Mantle cell lymphoma is the rarest of the subtypes, accounting for about 6% of all non-Hodgkin’s lymphoma cases in the United States and Europe. Lymphoid neoplasms of the urinary tract and male genital organs are relatively rare, accounting for less than 5% of extranodal lymphomas. We present a rare case of mantle cell lymphoma infiltrating the ureter causing pelvi-ureteric junction obstruction on tissue diagnosis.

**Case presentation:**

A 78-year-old Caucasian woman was referred to our department with right flank pain, pyrexia and features of a urinary tract infection. A nephrostogram revealed a grossly distended right pelvicalyceal system in a pelvi-ureteric junction obstruction pattern. She underwent an elective pyeloplasty after her acute management and the results of histological examination revealed mantle cell lymphoma.

**Conclusion:**

We describe a rare presentation of mantle cell lymphoma as a pelvi-ureteric junction obstruction. To the best of our knowledge, there has not been any previously published report of the above finding. Our patient had a history of a previous lymphoma but the aim of this manuscript is to highlight a possible presentation rather than determining whether the mantle cell lymphoma was *de novo* or a transformation from her previous splenic lymphoma with villous lymphocytes.

## Introduction

Mantle cell lymphoma (MCL) is one of the several subtypes of non-Hodgkin’s lymphoma. MCL is the rarest of the subtypes, accounting for about 6% of all non-Hodgkin’s lymphoma cases in the United States and Europe. It is the result of a malignant transformation of a B lymphocyte in the outer edge of a lymph node follicle, called the mantle zone. Those cells can spread through the lymphatics and blood to other lymph nodes or tissues such as the bone marrow, liver and gastrointestinal tract. MCL has the worst prognosis among lymphomas, with a median survival of approximately three to four years [1,2].

Lymphoid neoplasms of the urinary tract and male genital organs are relatively rare, accounting for less than 5% of extranodal lymphomas. We conducted a literature search of PubMed and MEDLINE using the keywords ‘ureter’, ‘mantle cell lymphoma’ and ‘lymphoma’. We found no reports of MCL involving the ureter.

We present a rare case of MCL infiltrating the ureter causing pelvi-ureteric junction obstruction (PUJO) on tissue diagnosis.

## Case presentation

**Figure 1:**
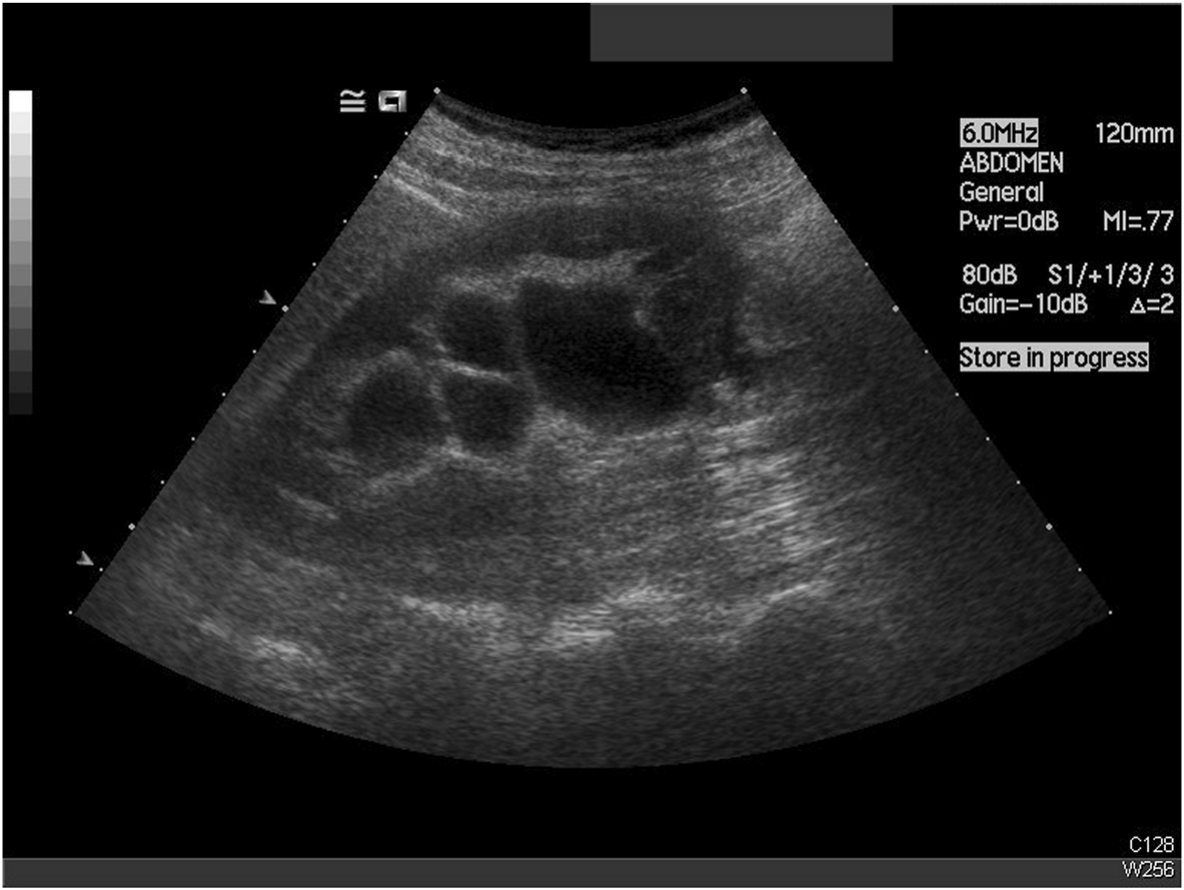
Ultrasound demonstrating hydronephrosis.

**Figure 2:**
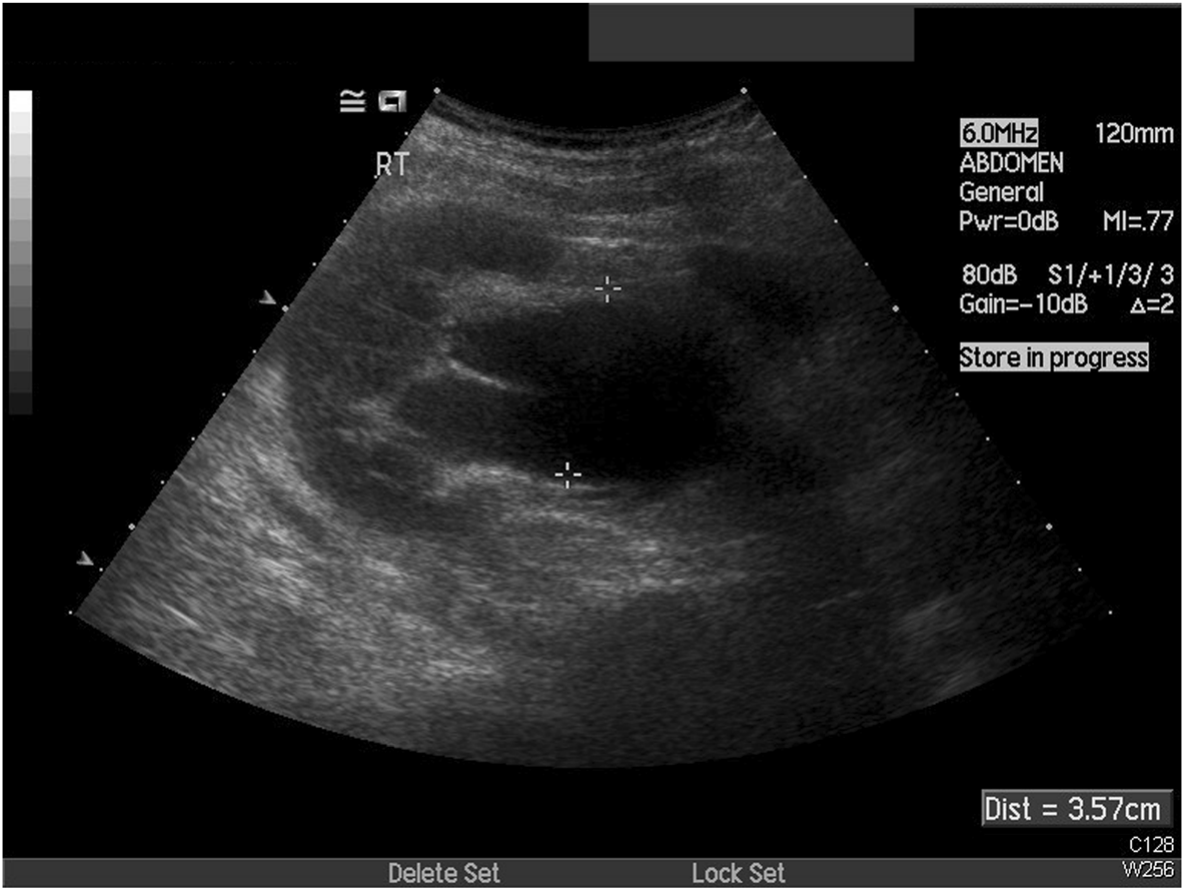
Ultrasound with hydronephrosis and dilated pelvis.

A 78-year-old Caucasian woman was referred to our department with right flank pain, pyrexia and features of a urinary tract infection. Part of her investigations included an emergency ultrasound scan. This suggested right hydronephrosis with debris, suggestive of infective material, but with no obvious obstruction, mass or calculi seen. In terms of her past medical history, she had a splenectomy 15 years ago for splenic lymphoma with villous lymphocytes (SLVL), and had undergone laparoscopic cholecystectomy, a left total hip replacement and hysterectomy. She had been under regular follow-up by the hematologists and been managed conservatively. Part of her follow-up with the hematologist included ultrasound and computed tomography scans, which suggested a long-standing right PUJO and some prominent lymph nodes in her porta hepatis (Figures 1 and 2).

**Figure 3:**
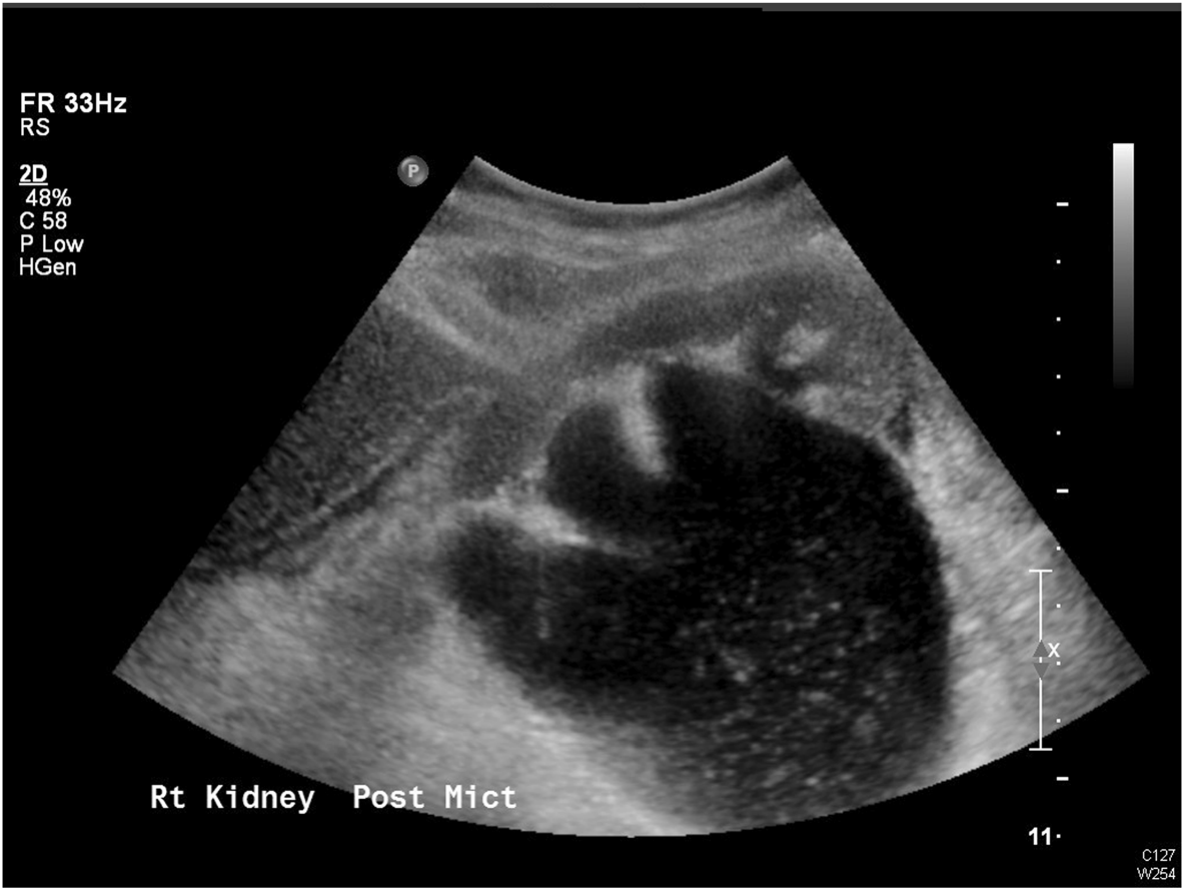
Persisting hydronephrosis on ultrasound with debris.

On the evening of admission, our patient underwent a right nephrostomy insertion under imaging guidance in view of the findings on her repeat ultrasound (Figure 3). Results of her biochemical studies reported a white blood cell count of 32.7×10^9^ cells/L), C-reactive protein level of 202mg/L and a creatinine level of 113μmol/L (with a baseline of 60μmol/L) (Table 1). We drained bloody urine full of pus that grew coliform bacteria, which we treated with intravenous antibiotics.

**Table 1:** Laboratory investigations

	**Day 0**	**Day 5 (Discharge)**
**Hemoglobin (g/dL)**	12.6	11.7
**White blood cell count (×10**^**9**^**/L)**	32.7	14.3
**Neutrophils (×10**^**9**^**/L)**	20.9	5.3
**Creatinine (μmol/L)**	113	64
**C-reactive protein (mg/L)**	202	107

Over the next few days, our patient recovered quite well and was discharged from hospital after a five-day stay.

**Figure 4:**
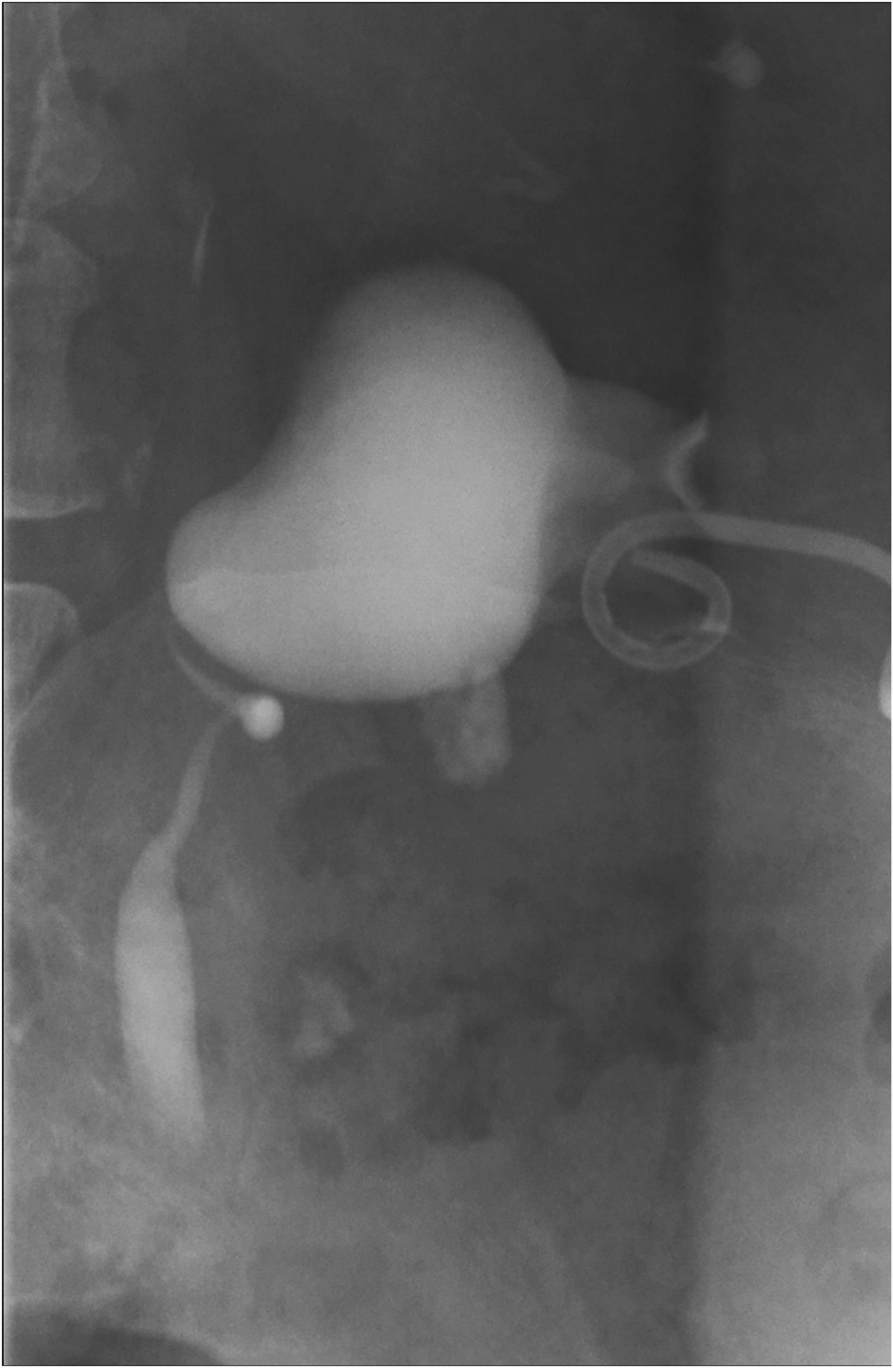
Nephrostogram showing the pelvi-ureteric junction obstruction pattern pattern.

In terms of her outpatient investigations, she underwent a dimercaptosuccinic acid renogram, which showed 52% function in her right kidney and 48% in her left. A nephrostogram revealed a grossly distended right pelvicalyceal system in a PUJO pattern. The contrast passed out in small aliquots and without obstruction into her bladder with just a little hold up over her iliac vessels (Figure 4).

Our patient underwent an elective laparoscopic pyeloplasty two months after being discharged. The procedure included a cystoscopy, which revealed an inflamed bladder wall with normal ureteric orifices, and a right retrograde that showed a normal ureter with narrowing and a kink at the right pelvi-ureteric junction, with contrast spill into her pelvis - typical of a PUJO. The pyeloplasty was uneventful and her 6Fr stent was removed at flexible cystoscopy four weeks after the operation.

**Figure 5:**
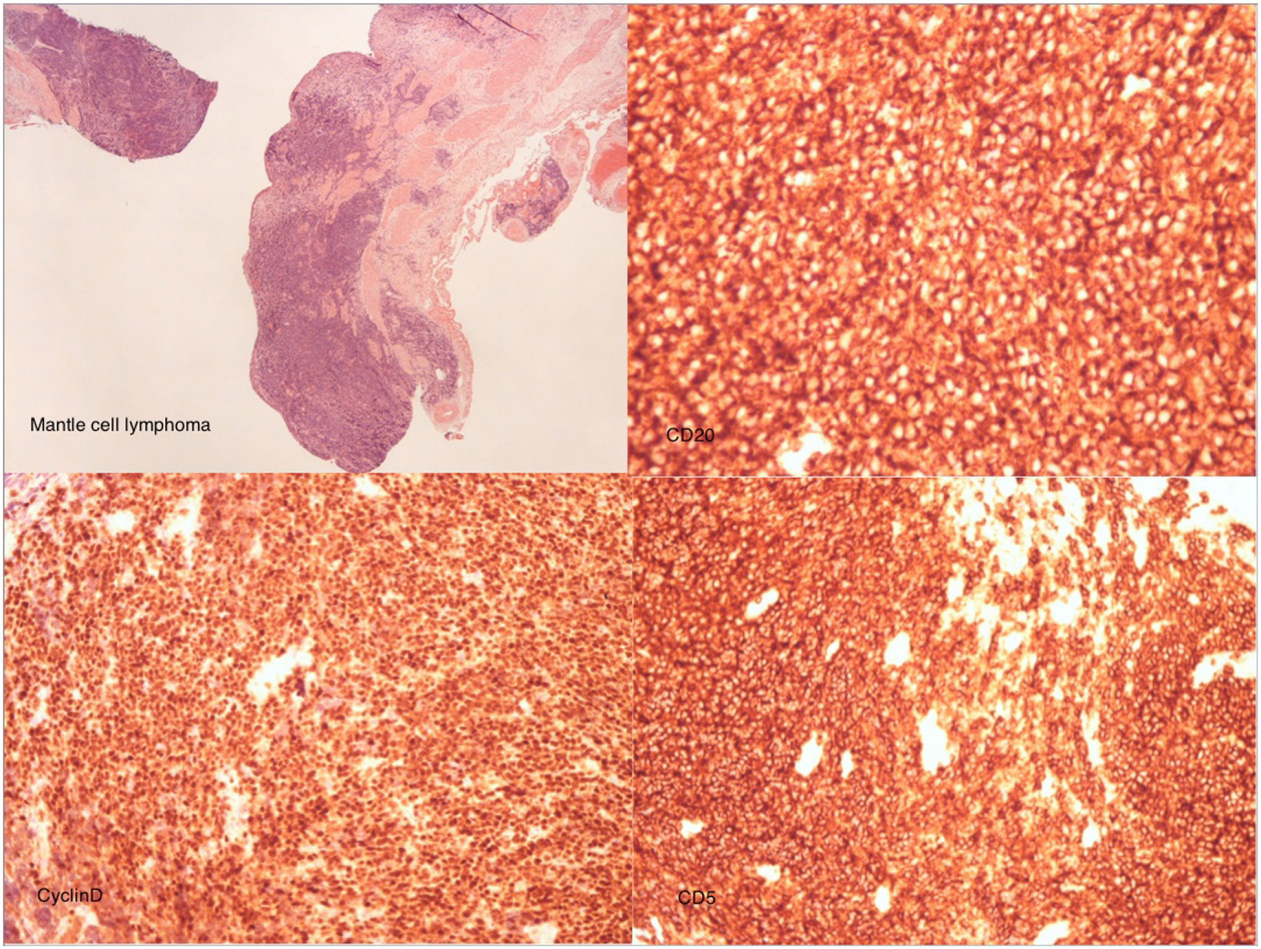
Histology slides: Mantle cell lymphoma, CD20, CyclinD and CD5 staining.

**Figure 6:**
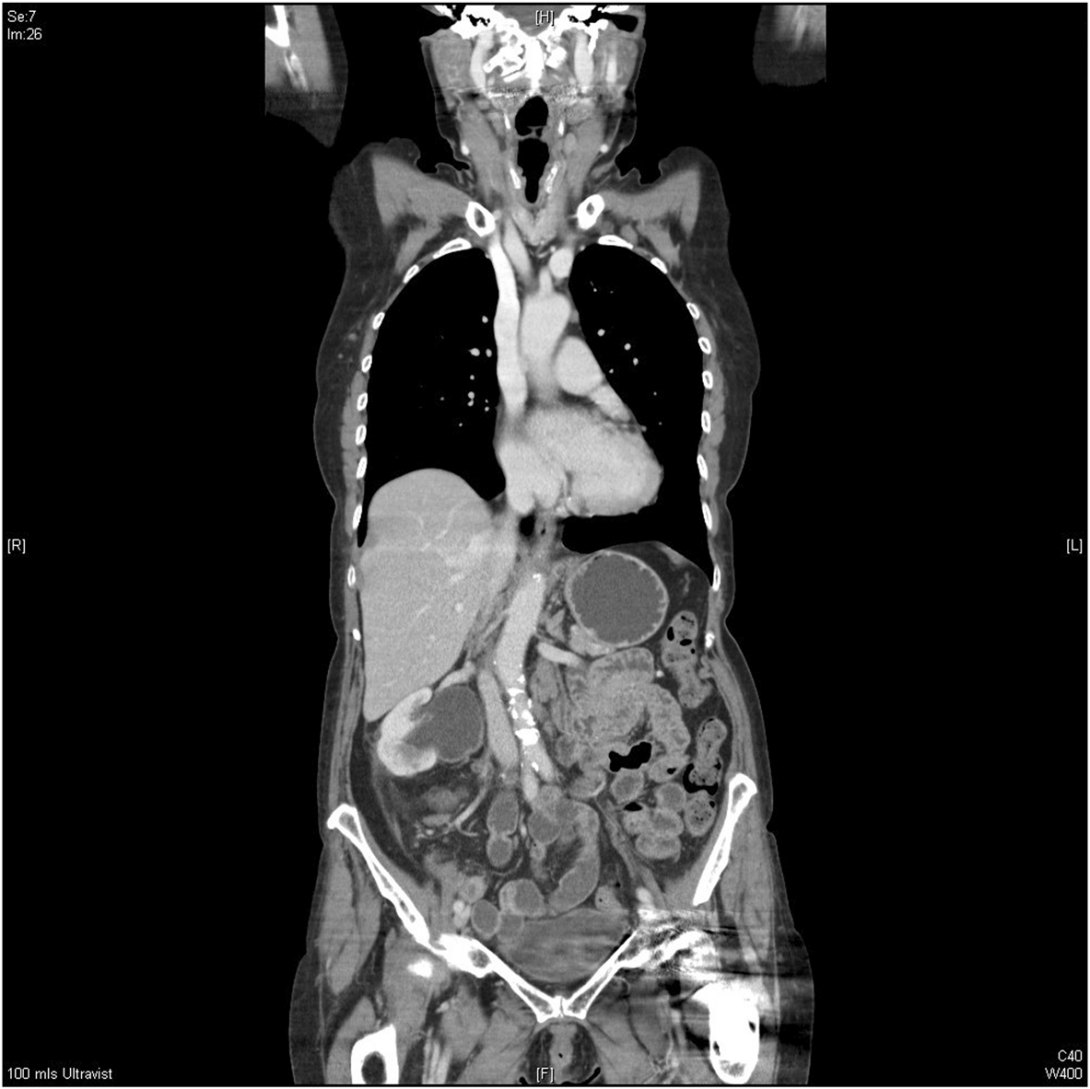
Computed tomography scan with persisting right PUJO and minor lymphadenopathy.

Histology of the specimen showed a dilated ureter with loss of surface urothelium in many areas. The submucosa, muscle and deeper tissues contained a patchy but heavy infiltrate of small lymphoid cells without any follicular structures. These cells showed widespread positivity for B-cell markers cluster of differentiation (CD) 20 and CD79a, staining with markers B-cell lymphoma 2 and cyclin D2, weaker diffuse staining with CD5 and a low proliferation fraction, less than 5%, with marker Ki67. These features are consistent with MCL (Figure 5). A recent computed tomography scan suggested persistence of her minor lymphadenopathy and continuing appearance of the right PUJO (Figure 6). An intravenous urogram was performed, with both kidneys concentrating contrast appropriately, with a pelvicalyceal system that was prominent on the right and evidence of appropriate drainage bilaterally.

Our patient has now been referred back to the hematologist for further management of her MCL and is undergoing rituximab, cyclophosphamide, doxorubicin, vincristine and prednisone chemotherapy treatment.

## Discussion

Lymphomas in the genitourinary tract are extremely rare, only a handful of small case series and isolated reports have been published describing their predominant sites and subtypes [3]. To the best of our knowledge, lymphoma infiltrating the ureter has only been mentioned by Schniederjan and Osunkoya in their case series [4]. MCL involving the ureter has not been described before.

MCL is a subtype of B-cell lymphoma, derived from CD5-positive antigen-naïve pregerminal center B-cells within the mantle zone that surrounds normal germinal center follicles. MCL cells generally over-express cyclin D1 due to a t(11:14) chromosomal translocation in the deoxyribonucleic acid (DNA). The cause is unknown and no inherited predisposition has been identified [5].

Ki-67 is an indicator of how fast cells mature: the lower the percentage, the lower the speed of maturity, and the more indolent the disease. In our scenario, our patient expressed less than 5% staining [6].

She had previously been diagnosed with SLVL, after which she had a splenectomy. Unfortunately, it was unclear how this diagnosis was made. She had been asymptomatic until her admission for pyonephrosis and follow-up scans after her splenectomy attributed her right hydronephrosis to a ‘congenital’ PUJO.

With good renal function demonstrated on her dimercaptosuccinic acid scan and her history of SLVL, one should be suspicious of attributing the finding to a congenital condition.

SLVL is a lymphoproliferative disorder characterized by the presence in the peripheral blood of atypical B-lymphocytes. Clinical features include massive splenomegaly and absence of peripheral lymphadenopathy. In our patient, an incidental finding of splenomegaly led to her having a splenectomy for SLVL [7].

In retrospect, we could argue that with good renal function on her previous imaging and with her history of SLVL, her right-sided hydronephrosis was secondary to the lymphoma process rather than to a congenital finding.

## Conclusion

We describe a rare presentation of MCL as a PUJO. To the best of our knowledge, there has not been any previously published report of the above finding. Our patient had a history of a previous lymphoma but the aim of this manuscript was to highlight a possible presentation rather than determining whether the MCL was *de novo* or a transformation from her previous SLVL.

## Consent

Written informed consent was obtained from the patient for publication of this case report and any accompanying images. A copy of the written consent is available for review by the Editor-in-Chief of this journal.

## Competing interests

The authors declare that they have no competing interests.

## Authors’ contributions

IN and RZ performed the main authorship and data collection. RZ reviewed the literature. TL revised the manuscript. All authors read and approved the final manuscript.
